# Prevalence and influencing factors of anemia among pregnant women across first, second and third trimesters of pregnancy in monitoring areas, from 2016 to 2020: a population-based multi-center cohort study

**DOI:** 10.1186/s12889-024-18610-x

**Published:** 2024-04-22

**Authors:** Yuting Qiao, Jiangli Di, Lina Yin, Aiqun Huang, Wei Zhao, Huanqing Hu, Sidi Chen

**Affiliations:** National Center for Women and Children’s Health, Chinese Center for Disease Control and Prevention, No.12, Dahuisi Road, Beijing, 100081 People’s Republic of China

**Keywords:** Anemia, Pregnant women, Pregnancy trimesters, Prevalence

## Abstract

**Objective:**

To assess the prevalence of anemia among pregnant women across their entire pregnancy and the factors affecting it in the monitoring areas.

**Methods:**

A total of 108,351 pregnant women who received antenatal health care and delivered from January 1, 2016 to December 31, 2020 in 15 monitoring counties of 8 provinces in the Maternal and Newborn Health Monitoring Program (MNHMP) of National Center for Women and Children’s Health (NCWCH) were selected as the study subjects. The anemia status among the subjects across their first, second and third trimester of pregnancy and the influencing factors were analyzed.

**Results:**

From 2016 to 2020, the prevalence of anemia at any stage during pregnancy in the monitoring areas was 43.59%. The prevalence of anemia among pregnant women across all three trimesters was 3.95%, and the prevalence of mild and moderate-to-severe anemia was 1.04% and 2.90%, respectively. Protective factors were living in the northern area (OR = 0.395) and being a member of an ethnic minority (OR = 0.632). The risk factors were residing in rural areas (OR = 1.207), with no more than junior high school education (OR = 1.203), having ≥ 3 gravidities (OR = 1.195) and multiple fetuses (OR = 1.478).

**Conclusions:**

Although the prevalence of anemia among pregnant women across all trimesters in the monitoring area was low, the severity of anemia was high. Since the prevalence of anemia among pregnant women across their entire pregnancy in the monitoring area is affected by many different factors, more attention should be paid to pregnant women living in rural areas, with low literacy, ≥ 3 gravidities and multiple fetuses for early intervention.

## Introduction

Anemia in pregnancy is a key contributor to maternal mortality and adverse pregnancy outcomes [1], affecting the health of more than 800 million women and newborns worldwide [2]. Studies have shown that anemia in pregnancy can increase the risk of various pregnancy complications such as hypertension, preeclampsia, premature rupture of membranes, postpartum hemorrhage and puerperal infection [3, 4], and that children born to anemic mothers are up to about 50% more likely to be anemic [5], which can lead to an increased risk of premature birth [6, 7], stillbirth [8], low birth weight [9], and perinatal death [10, 11]. Anemia among pregnant women throughout the entire pregnancy may be more harmful to both mother and baby. Currently, there are many relevant studies, but most of them focus on anemia occurring only once in the entire pregnancy or anemia at a certain stage of pregnancy, and there is a lack of research on anemia across the entire pregnancy.

This paper hopes to systematically monitor the hemoglobin level of pregnant women throughout pregnancy, to understand the status and influencing factors of anemia across the first, second and third trimesters of pregnancy, so as to provide a basis for the formulation of relevant policies and the evaluation of the effectiveness of the strategies implemented.

## Methods

### Data collection

The data of this study are derived from the Maternal and Newborn Health Monitoring System (MNHMS) set up by the National Center for Women and Children’s Health (NCWCH) for the MNHMP in 2013 [12]. The main aim of the MNHMP is to monitor the quality of maternal and infant’s health care prospectively and dynamically, and to improve the mothers and infants’ health. All pregnant women who reside for more than 6 months, receive antenatal care and give birth in the monitoring areas are the monitoring objects of the MNHMP. The MNHMS systematically collects a wide range of data about these women, including information on maternal characteristics, antenatal care (ANC) and delivery.

Maternal information including maternal residence address, age, ethnicity, education level, gravidity, parity, last menstrual period (LMP) is collected by qualified nurses in face-to-face interviews at the first prenatal examination. Other information about the results of ANC during pregnancy, such as Hemoglobin (Hb) value as well as the number of fetuses is collected through laboratory examinations and ultrasound examinations. All of the above information is required to be recorded and reported to the MNHMS promptly, completely and accurately. In order to ensure the quality of the data, the NCWCH provides specialized training for healthcare personnel, and organizes an expert group to conduct on-site supervision and quality control to standardize operation procedures of examinations and calibration of measurement in the monitoring area every year. Meanwhile, the MNHMS has set up logic and abnormal value error checking systems, and after the data have been reported, the data are verified and cleaned up by the level-by-level audit at the prefectural, provincial and national levels.

In order to maintain the continuity and integrity of the data, this study selected maternal case data from 8 provinces and 15 districts (counties) monitored from January 1, 2016 to December 31, 2020 in the MNHMS for analysis. The 8 provinces (with the selected 15 districts/counties) are: Sichuan (Gongjing and Rong county), Yunnan (Tonghai and Huaning), Hubei (Luotian and Huangmei), Hunan (Yueyang Lou and Yueyang), Hebei (Xinhua and Zhengding), Liaoning (Lishan), Fujian (Haican and Jimei) and Guangdong (Zijin and Longchuan). The research objects of this study were the monitoring objects in the above monitoring areas. The inclusion criterion for the research objects included (1) underwent antenatal examination and gave birth from January 1, 2016 to December 31, 2020; (2) the first antenatal examination was in the first trimester; (3) the records of Hb were complete across the first, second and third trimester of pregnancy. The exclusion criterions were (1) LMP was missing; and (2) there was no regular antenatal examination. Finally, a total of 108,351 pregnant women were included in the analyses of this study. According the formula for calculating the sample content of the estimated population rate from observational studies,we estimated that a sample size of 374 in each districts, increasing to 431 to allow for a 15% loss to follow-up, would have 90% power (2-tailed and at a 5% significance level) to estimate the prevalence of anemia among pregnant women in the monitoring area. After calculation, 6465 pregnant women should be investigated in 15 districts (counties) of 8 provinces, and the sample size of this study met the requirements.The collection of monitoring data of the MNHMP was approved by the Ethics Committee of the National Center for Women and Children’s Health, Chinese Center for Disease Control and Prevention (No.FY2015–007), and an ethical review is conducted every year (No.FY2018–14 and FY2019-12). This study was also approved by the Ethics Committee of the National Center for Women and Children’s Health, Chinese Center for Disease Control and Prevention (No.FY2022-05).

### Measure

1. Diagnostic criteria of anemia during pregnancy: In accordance with WHO diagnostic criteria [13], pregnant woman with Hb < 110 g/L detected at any time in the prenatal examinations was diagnosed as anemia during pregnancy. For pregnant woman who had multiple hemoglobin tests during pregnancy, severity of anemia was judged on the minimum HB value.

$$\mathrm{Prevalence\,of\,anemia\,during\,pregnancy}=\frac{\mathrm{Number\,of\,pregnant\,women\,with\,Hb\,}<\frac{110{\text{g}}}{{\text{L}}}\mathrm{detected\,any\,time\,during\,pregnancy}}{\mathrm{number\,of\,pregnant\,women\,with\,at\,least\,one\,Hb\,test\,during\,pregnancy\,}}\times\,100\mathrm{\%}$$

(2) Diagnostic criteria for the degree of anemia: mild anemia (100-109 g/L), moderate-to-severe anemia (≤ 99 g/L). Because the altitude of both Tonghai and Huanning in Yunnan Province is 1,600–2,000 m above sea level, the diagnostic criteria for degree of anemia degree after altitude correction are: mild (105-114 g/L) and moderate-to-severe (≤ 104 g/L).
(3) Based on WHO recommendations, the Guideline for Maternal Health Care Service (GMHCC) [14] defines the first trimester as up to 12^+6^ weeks of gestation, the second trimester as 13–27 + 6 weeks of gestation, and the third trimester as 28 weeks of gestation and beyond.
(4) Anemia among pregnant women across the entire pregnancy means that pregnant women were detected Hb < 110 g/L any time across the first, second and third trimester.

$$\mathrm{Prevalence\,of\,anemia\,among\,pregnant\,women\,across\,the\,entire\,pregnancy}=\frac{\mathrm{number\,of\,pregnant\,women\,with\,Hb\,}<\frac{110{\text{g}}}{{\text{L}}}\mathrm{detected\,any\,time\,in\,the\,first},\mathrm{\,secondand\,thirdst\,trimister}}{\mathrm{number\,of\,pregnant\,women\,with\,at\,least\,one\,Hb\,test\,in\,the\,first},\mathrm{\,second\,and\,third\,trimis\,}}\times\,100\mathrm{\%}$$

(5) According to the geographical location and economic development level and the division of the eastern, central and western regions of China of the *2021 China Health Statistical Yearbook*, Hebei, Fujian, Guangdong and Liaoning province represent the eastern region, Hunan and Hubei province represent the central region, and Sichuan and Yunnan province represent the western region.
(6) With the Qinling-Huaihe line as the boundary, Sichuan, Yunnan, Hubei, Hunan, Fujian and Guangdong are classified southern areas, and Hebei and Liaoning are classified as northern areas.

### Statistical analysis

The data set was imported from Excel into SAS9.4 for data cleaning. The database included the following information about each pregnant woman’s antenatal care: type of resident, education level, ethnicity, antenatal examination time, LMP, and gestational age, gravidity, parity, hemoglobin value, etc. Count data was described by frequencies (n) and composition ratios by percentages (%). The ${\chi }^{2}$ test was used for univariate analysis of the differences in the prevalence of anemia among pregnant women in each trimester in the monitoring areas. Binary logistic regression was used to analyze the factors affecting anemia in pregnant women in each trimester. With or without anemia was taken as the dependent variable, and the factors with statistically significance differences in the results of univariate analysis were taken as the independent variable. A stepwise method was used to screen the independent variables, with ${\alpha }_{inclusion}=0.05$ and ${\alpha }_{exclusion}=0.10$. The test level was $\alpha =0.05$, and a two-sided test was adopted. *P* < 0.05 was regarded as a statistically significant difference.

## Results

Among the 108,351 pregnant women, the majority of them (81.39%) came from the southern area. The age group was mainly concentrated in the group 18–34 years old (89.40%). The majority of the pregnant women (66.79%) had been pregnant more than once. Table 1 shows the detail.

**Table 1:** Demographic information of pregnant women in monitoring areas, from 2016 to 2020 (*N* = 108,351)

Variable	Number of subjects(n)	Composition ratio (%)	Variable	Number of subjects (n)	Composition ratio (%)
**Region**			**Education level**		
Eastern	59248	54.68%	Primary school or lower	3540	3.27%
Central	22661	20.91%	Junior high school	31317	28.90%
Western	26442	24.40%	Senior high school/technical school	31920	29.46%
**Area**			College or above	41574	38.37%
South	88191	81.39%	**Gravidity**		
North	20160	18.61%	1	35986	33.21%
**Residence**			2	43784	40.41%
Urban	46654	43.06%	≥ 3	28581	26.38%
Rural	61697	56.94%	**Parity**		
**Age (years old)**			0	48820	45.06%
< 18	701	0.65%	≥ 1	59531	54.94%
18 ~ 34	96871	89.40%	**Number of fetuses**		
≥ 35	10779	9.95%	Singleton	107482	99.20%
**Ethnicity**			Multiple	869	0.80%
Han	103431	95.46%			
Ethnic minorities	4920	4.54%			
**Type of residents**					
Local	103761	95.76%			
Non- local	4590	4.24%			

From 2016 to 2020, a total of 47,231 out of 108,351 pregnant women in the monitoring areas were diagnosed with anemia at some stage during pregnancy, with a prevalence of 43.59%. The prevalence of mild and moderate-to-severe anemia during pregnancy was 28.56% (30945/108,351) and12.71% (13,771/108,351), respectively. A total of 4,275 out of 108,351 pregnant women in the monitoring areas were detected with anemia across all trimesters of pregnancy. The prevalence of anemia among pregnant women across the entire pregnancy was 3.95%, with the prevalence of mild and moderate-to-severe anemia being 1.04% (1,130/108,351), and 2.90% (3,145/108,351), respectively.

Table 2 presents the prevalences of anemia at any stage during pregnancy and anemia across the entire pregnancy. These prevalences were different among pregnant women for region, area, age group, ethnicity, education level, type of resident and pregnancy status, and the differences were all statistically significant (all *P* < 0.05). Among pregnant women in the central regions, the prevalence of anemia during pregnancy was the lowest (37.5% vs 44.77% in eastern regions and 46.17% in western regions), while the prevalence of anemia across the entire pregnancy was the highest (6.12% vs 3.30% in the eastern regions and 3.53% in the western regions). In the southern area, the prevalence of anemia during pregnancy (41.91%) was significantly lower than that in the northern area (50.95%), but the prevalence of anemia across the entire pregnancy (4.42%) was significantly higher than that in the northern area (1.87%). The prevalence of anemia during pregnancy was the highest among the < 18 years old group (54.78% vs 43.48% in the 18–34 years old group and 43.89% in the ≥ 35 years old group), but the prevalence of anemia across the entire pregnancy was highest in the advanced age group (4.67% vs 4.46% in the < 18 years old group and 3.86% in the 18–34 years old group). Among women of Han ethnicity, the prevalence of anemia during pregnancy (43.24%) was significantly lower than among women of ethnic minorities (50.98%), but the prevalence of anemia across the entire pregnancy (3.98%) was significantly higher among Han than among ethnic minorities (3.15%).

**Table 2:** Univariate analysis of pregnant women with anemia

Variables	Anemia during pregnancy					Anemia across the entire pregnancy				
	**Anemia (n)**	**Non-anemia (n)**	**Prevalence (%)**	${\chi }^{2}$	***P***	**Anemia (n)**	**Non-anemia (n)**	**Prevalence (%)**	${\chi }^{2}$	***P***
**Region**										
Eastern	26527	32721	44.77	447.255	< 0.001	1955	57293	3.3	360.238	< 0.001
Central	8497	14164	37.5			1387	21274	6.12		
Western	12207	14235	46.17			933	25509	3.53		
**Area**										
South	36959	51232	41.91	545.889	< 0.001	3899	84292	4.42	282.869	< 0.001
North	10272	9888	50.95			376	19784	1.87		
**Residence**										
Urban	19372	27282	41.52	142.505	< 0.001	1646	45008	3.53	37.667	< 0.001
Rural	27859	33838	45.15			2629	59068	4.26		
**Age(years old)**										
< 18	384	317	54.78	36.596	< 0.001	14	300	4.46	16.709	< 0.001
18 ~ 34	42116	54755	43.48			3758	93500	3.86		
≥ 35	4731	6048	43.89			503	10276	4.67		
**Ethnicity**										
Han	44723	58708	43.24	114.311	< 0.001	4120	99311	3.98	8.598	0.003
Ethnic minorities	2508	2412	50.98			155	4765	3.15		
**Education**										
Primary school and lower	1766	1774	49.89	229.284	< 0.001	147	3393	4.15	161.295	< 0.001
Junior high school	14176	17141	45.27			1462	29855	4.67		
Senior high school/technical school	14274	17646	44.72			1417	30503	4.44		
College and above	17015	24559	40.93			1249	40325	3.00		
**Type of residents**										
Local	45094	58667	43.46	17.159	< 0.001	4080	99681	3.93	1.16	0.282
Non-local	2137	2453	46.56			195	4395	4.25		
**Gravidity**										
1	14710	21276	40.88	284.179	< 0.001	1277	34709	3.55	45.829	< 0.001
2	18954	24830	43.29			1690	42094	3.86		
≥ 3	13567	15014	47.47			1308	27273	4.58		
**Parity**										
0	20586	28234	42.17	73.234	< 0.001	1845	46975	3.78	6.486	0.011
≥ 1	26645	32886	44.76			2430	57101	4.08		
**Number of fetuses**										
Singleton	46752	60730	43.5	47.362	< 0.001	4228	103254	3.93	4.948	0.026
Multiple	479	390	55.12			47	822	5.41		

Variables with statistical significance in univariate analysis were introduced into multivariate logistic regression using the stepwise method. In the end, there were 6 variables in the equation: region, urban or rural areas, ethnicity, education level, gravidity, number of fetuses.

Table 3 shows the results of multi-factor logistic regression analysis. Living in the northern area and being of an ethnic minority were protective factors for anemia across the entire pregnancy. Pregnant women residing in the northern area had a lower risk of anemia across the entire pregnancy compared to those residing in the southern area (OR = 0.395, 95% CI: 0.354–0.441). Pregnant women from ethnic minorities had a lower risk of anemia across the entire pregnancy than ethnic Han pregnant women (OR = 0.632, 95% CI: 0.536–0.747).

**Table 3:** Multi-factor logistic regression analysis of anemia across the entire pregnancy

Variables	*β*	*S.E*	*Waldχ*2	*P*	*OR*(95%*CI*)
**Area**					
South					1.000
North	-0.928	0.056	277.588	< 0.001	0.395(0.354, 0.441)
**Residence**					
Urban					1.000
Rural	0.188	0.036	27.381	< 0.001	1.207(1.125, 1.295)
**Ethnicity**					
Han					1.000
Ethnic minorities	-0.458	0.085	29.323	< 0.001	0.632(0.536, 0.747)
**Education**					
Primary school and lower					1.000
Junior high school	0.185	0.089	4.289	0.038	1.203(1.010, 1.433)
Senior high school/technical school	0.163	0.090	3.296	0.069	1.177(0.987, 1.404)
College and above	-0.111	0.093	1.427	0.232	0.895(0.746, 1.074)
**Gravidity**					
1					1.000
2	0.024	0.038	0.379	0.538	1.024(0.950, 1.104)
≥ 3	0.178	0.043	17.516	< 0.001	1.195(1.099, 1.299)
**Number of fetuses**					
Singleton					1.000
Multiple	0.391	0.152	6.648	0.009	1.478(1.098, 1.988)

Living in a rural area, having no more than a junior high school education, with ≥ 3 gravidities, and having multiple fetuses were risk factors for anemia across the entire pregnancy. Compared with those living in urban areas, pregnant women living in rural areas had a higher risk of anemia across the entire pregnancy (OR = 1.207, 95%CI: 1.125 ~ 1.295). Pregnant women with ≥ 3 gravidities were more likely to have anemia across the entire pregnancy than those with 1 gravidity (*OR* = 1.195, 95%*CI*: 1.099 ~ 1.299). Pregnant women with multiple fetuses had a higher risk of anemia across the entire pregnancy than women with a singleton fetus (OR = 1.478, 95%CI: 1.098 ~ 1.988).

## Discussion

The results of this study showed that the prevalence of anemia at any stage during pregnancy in the monitoring areas was 43.59% in 2016–2020. The Report on Nutrition and Chronic Disease Status of Chinese Residents in 2020 [15] reported that the prevalence of anemia during pregnancy in China was 13.6% in 2015–2017, which was much lower than the results of this study. In 2021, a bibliometric study that included 109 papers was conducted by researchers from Peking Union Medical College on the prevalence of anemia during pregnancy in China [16]. It found that the prevalence of anemia during pregnancy in China ranged from 10 to 40%. However, because 93.6% of the included studies were cross-sectional studies, and many cross-sectional surveys did not systematically observe Hb levels throughout pregnancy, the authors believed that the results of the study underestimated the prevalence of anemia during pregnancy in China. Our study is based on data obtained through continuous monitoring of all of the pregnant women in the area throughout the entire gestation period. It systematically monitored the anemia situation at multiple points during pregnancy, so the anemia prevalence rate obtained is expected to be more objective and accurate.

According to the WHO’s criteria based on the prevalence for the severity of an anemia problem, an anemia prevalence of 5–19.9% is a mild public health problem, 20–39.9% is a moderate public health problem, and ≥ 40% is the standard of a severe public health problem [17]. It's obvious that anemia among pregnant women in our monitoring areas has become a severe and serious public health problem. Therefore, it is still a big challenge for the health authorities in these monitoring areas to achieve the goal proposed by the Action for a Healthy China (2019–2030), namely anemia prevalence among pregnant women of less than 14% in 2022 and less than 10% in 2030.

From 2016 to 2020, a total of 4,275 pregnant women in the monitoring areas were detected with anemia across the first, second and third trimesters. The prevalence of anemia among pregnant women across their entire pregnancy was 3.95%, and the prevalence of moderate-to-severe anemia (2.90%) was higher than that of mild anemia (1.04%). From the results of this study, the prevalence of mild anemia (28.56%) during pregnancy was much higher than that of moderate-to-severe anemia (12.71%), suggesting that the pregnant women with anemia across the entire pregnancy had a more severe anemia. There are two possible reasons for a greater severity of anemia: on the one hand, the anemia suffered by this group of pregnant women may be refractory anemia itself; on the other hand, these patients may not have received effective intervention after falling ill in the first trimester, resulting in anemia throughout the pregnancy with a greater severity.

The results of this study showed that the prevalence of anemia among pregnant women across their entire pregnancy in the south was higher than that among pregnant women in the north, which contrasts with our finding that the prevalence of anemia in any trimester during pregnancy in the south (41.91%) was lower than that in the north (50.95%). It is also inconsistent with the findings of another study of our center [12] and other studies [18] that the prevalence of anemia during pregnancy in the southern area was lower than that in the north.

Another study conducted by our center in the monitoring area [19] showed that the rate of use of an iron supplement by pregnant women after anemia was significantly higher in the north (42.1%) than in the south (34.1%). This may be due to the fact that although the prevalence of anemia during pregnancy in the north was higher than that in the south, the prevalence of anemia across the entire pregnancy in the south was higher than that in the north. Another reason for this result may also be that there is a high incidence of thalassemia in the south [20], and Guangdong, Yunnan and Sichuan provinces [21], as high incidence areas, are all in our scope of surveillance.

Thalassemia is an autosomal recessive genetic disease with high prevalence, which is difficult to treat [22–24]. Therefore, it is very likely to last throughout pregnancy, possibly making the anemia prevalence among southern pregnant women higher than that of the northerners across all trimesters.

The results of that study also showed that the prevalence of anemia among rural pregnant women was higher than among women urban areas across the entire pregnancy, which is consistent with the findings of this study and the other studies in which the prevalence of anemia during pregnancy was higher in rural areas than in urban areas [15, 25]. The results of another study conducted by our center in the monitoring area [19] showed that the rate of iron intake through supplementation during pregnancy (43.7%), consumption of red meat during pregnancy (91.0%) and consumption of animal blood or livestock liver (90.9%) among pregnant women undergoing prenatal examination in health care institutions at county level and above was much higher than that of pregnant women at township and village health centers (29.8%, 9% and 9.1%, respectively). The proportion of doctors in provincial and municipal hospitals who recommended iron supplementation during pregnancy (61.5% and 47.5% respectively) was also much higher than that of doctors in county and township level hospitals (39.6% and 23.5% respectively). It is suggested that the reasons why the prevalence of anemia both at any stage during pregnancy and across the entire pregnancy are higher in rural areas than in urban areas are as follows. On the one hand, it may be due to the insufficient compliance of rural women with anemia prevention and control measures; on the other hand, doctors in urban areas may pay more attention to anemia during pregnancy than doctors in rural areas, and the intervention and treatment measures taken may be more standardized.

More than 3 gravidities and multiple fetuses were risk factors for anemia across the entire pregnancy. The higher the number of pregnancies or parity, the higher the prevalence of anemia across the entire pregnancy. Pregnant women with more gravidities and parities and a higher number of fetuses during their pregnancies had a higher prevalence of anemia across the entire pregnancy. As an Ethiopian longitudinal study on the effect of pregnancy on hemoglobin levels [26] showed, multiple fetuses can reduce maternal hemoglobin levels. Therefore, in terms of parity, this result may be due to anemia not being diagnosed and treated in a timely manner during the previous pregnancy. Another possible scenario is: if iron stores are inadequate, a subsequent pregnancy results in an increased probability of anemia and difficulty in correcting it. These circumstances suggest that more attention should be paid to prenatal examination in the process of multiple fetuses and parturition, timely detection and correction of anemia, and prevention of further aggravation of anemia.

This study also has the following limitations: (1) This study only monitored the anemia status of pregnant women and did not distinguish the types of anemia, so the results of the study could not be interpreted thoroughly; (2) The study subjects are only from 15 monitoring counties (districts) in 8 provinces, and the regional and geographical representation needs to be strengthened. Despite the above limitations, this study has several real strengths. It is based on population, with large samples, from multiple regions, and follows all the subjects throughout pregnancy. Thus, the results are more valuable than cross-sectional studies from hospitals or populations.

## Conclusions

In summary, the prevalence of anemia at any point during pregnancy was higher in the monitoring area, and the degree of anemia was more severe among pregnant women who were anemic across all trimesters. Therefore, it is necessary to implement comprehensive prevention and control measures based on the occurrence and influencing factors of anemia among pregnant women. For example, health education strategies are carried out, enhancing dietary iron and folic acid intake through the Health Belief Model [27]. Specifically, more attention should be paid to pregnant women who live in rural areas, have more than 3 gravidities, and have multiple fetuses. Health workers need to intervene as soon as possible to reduce the incidence of anemia in pregnant women, so as to reduce the health impacts on the mother and child.

## Data Availability

The datasets generated and analyzed during the current study are not publicly available due to privacy restrictions but are available from the corresponding author on reasonable request.

## Abbreviations

MNHMS: Maternal and Newborn Health Monitoring System
NCWCH: National Center for Women and Children’s Health
ANC: Antenatal Care
LMP: Last Menstrual Period
Hb: Hemoglobin
WHO: World Health Organization
GMHCC: Guideline for Maternal Health Care Service
